# Fabrication and optimization of phospholipids-based phase separation *in-situ* gel loaded with BMP-2 nanosized emulsion for bone defect

**DOI:** 10.3389/fphar.2023.1286133

**Published:** 2023-10-17

**Authors:** Mohammed Alissa, Ahmed Hjazi, Ghadah S. Abusalim, Ghfren S. Aloraini, Suad A. Alghamdi, Waleed Y. Rizg, Khaled M. Hosny, Deena M. Bukhary, Hanaa Alkharobi

**Affiliations:** ^1^ Department of Medical Laboratory Sciences, College of Applied Medical Sciences, Prince Sattam Bin Abdulaziz University, Al-Kharj, Saudi Arabia; ^2^ Department of Pharmaceutics, Faculty of Pharmacy, King Abdulaziz University, Jeddah, Saudi Arabia; ^3^ Center of Innovation in Personalized Medicine (CIPM), 3D Bioprinting Unit, King Abdulaziz University, Jeddah, Saudi Arabia; ^4^ Department of Pharmaceutics and Industrial Pharmacy, Faculty of Pharmacy, Beni-Suef University, Beni-Suef, Egypt; ^5^ Department of Pharmaceutics, College of Pharmacy, Umm Al-Qura University, Makkah, Saudi Arabia; ^6^ Department of Oral Biology, Faculty of Dentistry, King Abdulaziz University, Jeddah, Saudi Arabia

**Keywords:** optimization, bone morphogenetic protein, nanoemulsion, phospholipids-based phase separation *in-situ* gel, l-optimal co-ordinate

## Abstract

**Introduction:** The health, development, and/or survival of a newborn can be impacted by congenital abnormalities such as cleft lip (CLP) and palate, one of alveolar bone defects that emerge thru pregnancy. Therefore, the primary purpose of this study is to use phospholipids-based phase separation *in-situ* gel (PPSG) in combination with bone morphogenetic protein-2 nanoemulsion (BMP-2-NE) to aid repairing alveolar bone defects.

**Methods:** To investigate how formulation parameters, such as the concentrations of BMP-2 aqueous solution, LauroglycolTM FCC, and Labrafac PG oil, affect NE qualities including droplet size and stability index, an l-optimal co-ordinate exchange statistical design was opted. Injectable PPSG with the best NE formulation was tested for viscosity characteristics, gel strength, water absorption, and *in-vitro* BMP-2 release. In rabbits, the percentage of BMP-2 that was still in the maxilla after 14 days was assessed.

**Results:** Collected results revealed that the droplet size and stability index of optimal NE were discovered to be 68 2.0 nm and 96 1.3%, respectively. When mixed with water, optimal BMP-2 NE loaded PPSG became viscous and reached a gel strength of 41 s, which is adequate for injectable *in-situ* gels. In comparison to BMP-2 solution loaded *in-situ* gel, the *in-vivo* studies indicated that the newly created BMP-2 NE loaded PPSG produced a sustained and controlled release of BMP-2 that continued for 336 h (14 days). Further, 8% of the BMP-2 was still entrapped and not completely dissolved after 14 days, thus, created formulation allowed a higher percentage of BMP-2 to remain in rabbits’ maxilla for longer time.

**Conclusion:** PPSG that has been loaded with BMP-2 NE may therefore be a promising, fruitful, and less painful paradigm for the noninvasive therapy of CLP with significant effect and extended release.

## 1 Introduction

Congenital malformations are conditions that are present at birth, develop in intrauterine life, and can affect the newborn’s health, growth, and/or survival. Cleft lip and palate (CLP) is a very common birth defect all over the world (DeSilva et al., 2016). It is an abnormality that predominantly affects facial structures, especially maxillary-mandibular structures (Falcini et al., 1996; Burnham et al., 2007). The management of CLP is difficult, because compensating for it demands a comprehensive multidisciplinary strategy involving several surgeries and extended orthodontic management (Evans, 2004). Bone grafting surgery for the accompanying alveolar abnormalities has a substantial place in CLP treatment (Gimbel et al., 2007).

The most employed grafting fabric is bone obtained from the iliac crest, but its use is associated with many disadvantages, such as a high incidence of morbidity at the site of origin, confined giver tissues, the possibility of resorption, and scarring due to the extent of the surgery (Swan and Goodacre, 2006). Other grafting methodologies utilize autologous cells to rejuvenate alveolar cleft bone or osteoinductive proteins (Hibi et al., 2006; Sawada et al., 2009) such as bone morphogenetic proteins (BMPs).

BMPs belong to the transforming growth factor superfamily and can act as osteoinductive elements, which can prompt the differentiation of osteoblasts from mesenchymal cells (Mori et al., 2000; Hassan et al., 2003). The efficiency of BMPs in spurring bone development was demonstrated in preceding investigations using many models and several clinical implementations in orthopedics or oral and maxillofacial surgery (Hassan et al., 2010). The prolonged release of growth proteins at the intended site of an implant was achieved by using various delivery systems composed of biodegradable materials that carried biologically safe growth factors (Herford and Boyne, 2008). The materials used in these delivery systems offered a felicitous way of delivering growth factors, including lactide-glycolide copolymers, tricalcium phosphate, collagen, and gelatin hydrogels (Sun et al., 2017). However, the delivery systems were fabricated in a solid state and therefore could only be implanted in the CLP through conventional surgical operations (Kowalczewski and Saul, 2018).

Nanotechnology is defined as the manipulation of materials at the nanoscale level (Salem et al., 2022b). Investigations into nanotechnology caught the attention of researchers in several countries and large corporations and led to investments in the field, which developed swiftly (Ali et al., 2021; Hussein et al., 2022). Nanosized materials have unprecedented physical, chemical, and biological merits and may improve or alter drug characters (Alhakamy et al., 2022; Salem et al., 2022a). Nanoemulsions (NEs) are progressive delivery paradigms that are convenient for boosting drug properties such as solubility and stability (Rizg et al., 2021). They are formed usually of oil globules in the nanosize range that are incorporated into an admixture of a surfactant and a co-surfactant, and they are largely investigated as delivery platforms for several active agents (Hosny et al., 2021b). NEs have several traits that are superior to traits of other dosage forms, such as 1) a fast absorption rate with few variations; 2) a protective action against oxidation or hydrolysis; 3) the ability to carry hydrophobic or hydrophilic agents; 4) an augmented bioavailability; 5) few side effects and little toxicity or irritation, especially when the skin or mucosa is the administration site; and 6) prolonged drug release and enhanced permeation (Hosny et al., 2021a). Some of these advantages are ascribed to the nanosize of the globules and others to the type of surfactant and co-surfactant used (Hosny et al., 2021c).

Although proteins and peptides have attracted the attention of many investigators because of their considerable therapeutic efficiency, their delivery is greatly restricted by their low T_50_, swift disintegration, and rapid removal (Zhang et al., 2015). Therefore, it was suggested that they be incorporated into a phospholipid-based phase separation gel (PPSG), which is easily injected due to its low initial viscosity and its ability to change into an *in-situ* insert after being placed in a watery medium (Zhang et al., 2019). *In-situ* implants are auspicious paradigms due to their injectability and capacity to form drug depots (Hatefi and Amsden, 2002; Dai et al., 2009). In addition, PPSGs are attractive *in-situ* implants due to their biocompatibility, low toxicity, and high availability of phospholipids. Further, phase transition *in-situ* gels based on phospholipids demonstrated excellent clinical promise to solve the shortcomings of other *in-situ* gels such as those based on PLGA which usually acquire certain toxicity. (Vintiloiu and Leroux, 2008; Helgeson et al., 2012; Kempe and Mäder, 2012).

Attaining the most information with the fewest trials, defining the interactions between variables, and clarifying the origins of experimental errors were the chief goals of this statistical experimental design (Alkhalidi et al., 2018). An additional benefit of such a design is that it can be planned precisely and requires the use of statistical equations. Such rigor obliges investigators to be accurate in setting up their study goals and choosing the tests to be performed to fulfill those goals. Additionally, the design can focus on the composition of the best formulation, its conductance, and its development on a large scale (Hosny et al., 2020). Experimental designs are, in some ways, economical, as they often offer the most acceptable solution for a formulation (Salem et al., 2020).

The leading objective of the present study was to formulate a novel injectable *in-situ* gel, recombinant human bone morphogenetic protein-2 in a nanoemulsion (BMP-2-NE) thru experimental design approach for the handling of a CLP with no surgical intervention. Through utilization of specific type of phospholipids which are virtually insoluble in aqueous solution but soluble in ethanol. As a result, if subjected to an aqueous solution, the phospholipids that were dispersed in ethanol will solidify and turn into a drug deposit. Here, we seek to take use of this characteristic to create an easily implantable *in-situ* graft (PPSG), and to examine its *in-vivo* and *in-vitro* phase switch features.

## 2 Materials and methods

### 2.1 Materials

Recombinant human BMP-2 (rhBMP-2) was obtained from Creative Biomart (Shirley, NY, United States). Phospholipid S100 was obtained from Lipoid GmbH (Ludwigshaven, Germany). Labrafac PG, Lauroglycol FCC, and Peceol (glycerol monooleate) were generously gifted by Gattefosse (Saint-Priest, France). Absolute ethanol was purchased from Sigma Chemical Company (St. Louis, MO, United States). All other chemicals and reagents were of HPLC grade and utilized with no further purification.

### 2.2 Methods

#### 2.2.1. Fabrication and optimization of BMP-2‒loaded NE as per mixture design

BMP-2‒loaded NE was fabricated using the I-optimal coordinate-exchange quadratic mixture design with Design-Expert software (version 13.0.7.0, Stat-Ease, Inc., Minneapolis, MN, United States). The experimental design checked the influence of certain factors, such as the percentages of BMP-2 aqueous solution (A), surfactant Lauroglycol FCC (B), and Labrafac PG oil (C); the used percentages were 10%–20%, 20%–40%, and 40%–70%, respectively. The three independent variables were mixed with varying ratios while keeping the total concentration at 100%. The average globule size (Y_1_) and stability indicator (Y_2_) were chosen as the measured variables. Fourteen formulations were created randomly. The chosen factors and their levels in every formulation are shown in Table 1. The associations between the independent variables and the dependent variables was further examined using regression equations and the statistical analysis methodologies of the Design-Expert software. All formed nanosuspensions were evaluated for their appearance and capacity for emulsification. The formulation having the smallest droplet size and best stability index was selected as the optimal formulation and subsequently utilized for developing a BMP-2-NE *in-situ* gel.

**TABLE 1:** Experimental outline of mixture design (showing levels of indpendent variables and dependent variables).

Indpendent variables	Level		Dependent variables	Aim
	Low	High		
BMP-2 aqueous solution level (A)	0.1	0.2	Mean globule size (Y_1_) Stability index (Y_2_)	Minimize Maximize
Lauroglycol FCC surfactant level (B)	0.2	0.4		
Labrafac PG oil level (C)	0.4	0.7		

#### 2.2.2 Preparation of BMP-2-NEs

NEs were developed by blending the Lauroglycol FCC emulsifier and a BMP-2 aqueous solution (100 μg/ml of BMP-2 in saline phosphate buffer) to form the aqueous phase. The dispersed aqueous phase was added dropwise to the oily continuous phase (i.e., Labrafac PG oil), and the mixture was agitated with a probe sonicator for 3 min with 5-s pulses (i.e., 5 s off and 5 s on) with a 40% amplitude to fabricate a water-in-oil emulsion (Hosny et al., 2021c).

#### 2.2.3 Assessment of the BMP-2-NEs

##### 2.2.3.1 Emulsification capacity

The speed of emulsification and the translucence of the BMP-2-NEs were visually examined to assess the NE competence (Rizg et al., 2021).

##### 2.2.3.2 Droplet size assessment

A volume of each NE was diluted with Peceol (ratio 1:10 v/v) to avoid the multiple scattering effect and reduce the influence of the samples’ viscosity. The dynamic light-scattering technique (Zetatrac, Microtrac, Montgomeryville, PA, United States) was applied to the samples to measure the NEs’ droplet size and polydispersity index and emulate the sizes of different samples (Rizg et al., 2021).

##### 2.2.3.3 Stability studies

The first step was to inspect the hot-cold stability of all of the NE formulations. The formulations were kept for 48 h at 4°C and then at 40°C for another 48 h. This cycle was replicated three times, and the formulations were then examined visually for evidence of instability. Then, the accelerated freeze–thaw stability was evaluated by applying diverse temperatures to affirm the thermodynamic stability of the NEs (Rizg et al., 2021). During this study, the droplet size was first assessed, and then the samples underwent three sequential freeze–thaw cycles (freezing at −25°C for approximately 24 h and thawing at 25°C for another 24 h). The droplet size was estimated after the successive cycles. The NEs’ stability indices were attained by rapproaching the final oil globules size with the former one, utilizing the equation below (Hosny et al., 2021b):

$$\text{Stability index}=\left(\left[\text{Initial size}-\text{Change in size}\right]/\text{Initial size}\right)\times 100$$ (1)

#### 2.2.4 Optimization of BMP-2-NEs

As per the goals listed in Table 1, the optimization of the BMP-2-NEs was performed. The optimization was done using 20% BMP-2 aqueous solution (A), 40% surfactant Lauroglycol FCC (B), and 40% Labrafac PG oil (C). The smallest droplet size and the largest stability index were chosen as the dependent variables for the optimal formulation.

#### 2.2.5 Fabrication and depiction of the optimal BMP-2-NE‒loaded *in-situ* gel

A phospholipid-based phase separation *in-situ* gel (PPSG) system was selected for transforming the optimal BMP-2-NE dispersion into an NE *in-situ* gel. In short, the gel base was developed by mixing Phospholipid S100 and Peceol (glycerol monooleate) in a 2:1 (w/w) ratio. Then, 10 ml absolute ethyl alcohol was added to this mixture using a magnetic stirrer at 800 rpm for 2 h at 25°C until a clear homogenous blend was formed. The optimal BMP-2-NE was added to the *in-situ* gel base and sonicated for 10 min in a water bath sonicator to create the BMP-2-NE‒loaded *in-situ* gel (10 μg/ml) (Hassan et al., 2016).

##### 2.2.5.1 Viscosity measurement

To obtain a gel, 30% phosphate buffered saline (PBS, 0.01 M, pH 7.4) was fully mixed with the optimal formulation loaded in the *in-situ* gel solution (Hassan et al., 2016). The viscosity was estimated at 25°C using a DV-2 T rotary viscometer (Brookfield, Middleboro, MA, United States) equipped with a No. 18 spindle at a speed of 20 rpm before and after the addition of PBS. The measurements were repeated three times and each replicate of the sample was assigned a label of A, B, or C. Following gelation, the samples were relabeled A*, B*, or C* (Abdelbary et al., 2017).

##### 2.2.5.2 Gel strength evaluation

Five grams of the optimal formulation loaded in the *in-situ* gel was transformed into a gel by mixing it with 1 ml of PBS (0.01 M, pH 7.4), as previously described. Then, a 3.5 g (0.7-cm in diameter) weight was placed on the gel’s surface. The gel strength, which indicates the *in-situ* gel viscosity based on the physiological conditions, was evaluated by determining how long, in seconds, it took for the weight to sink 3 cm into the gel (Hassan et al., 2016).

##### 2.2.5.3 Water absorption

A dialysis bag with about 1 g of BMP-2-NE‒loaded *in-situ* gel solution was dipped into 400 ml PBS (pH 7/4) and slowly mixed with the rest of the milieu using a magnetic stirrer at 200 rpm. At certain intervals (i.e., 5, 15, 30, 60, 120, 240, and 480 min), the gel was carefully removed and weighed. Then, an anhydrous methanol that was five times the weight of the sample was added to resolve the gel by ultrasonication. The water content of the methanol‒gel solution was assessed using the V20 Volumetric Karl Fischer moisture analyzer (Mettler Toledo, Columbus, OH, United States) with an injection volume of 200 μL. The concentration of water could be calculated depending on the dilution factor, and the proportion of water content to the dialyzed gel weight could also be determined (Hoshino et al., 2019).

##### 2.2.5.4 *In-vitro* release behavior of optimal formulation

A 5-ml specimen of the optimized BMP-2-NE‒loaded *in-situ* gel was placed in a dialysis bag and then into a beaker with 100 ml PBS (pH 6.8) at 37°C ± 1°C. The mixture was magnetically stirred at a speed of 50 rpm for 120 h. Samples were withdrawn at defined time intervals, and the BMP-2 content was estimated using a spectrophotometer at 280 nm (Jasco V530, Japan). The BMP-2 aqueous dispersion *in-situ* gel was used as a control in this experiment (Abdelbary et al., 2017).

##### 2.2.5.5 Evaluation of the *in-vivo* behavior of the optimal formulation

The *in-vivo* study was performed according to the institutional guidelines of the Animal Ethics Committee of Cairo Agriculture for Experimental Animals, Cairo, Egypt, Approval No. 91-10-22). Seventy-two 7-week-old male New Zealand White rabbits of 2.5–3.0 kg in weight were divided into three groups. They were injected subcutaneously in the maxilla with 0.5 mL of three different formulations: 1) the BMP-2 hydrogel developed by mixing the protein in 1% hydroxypropyl cellulose solution, 2) the optimized BMP-2-NE‒loaded *in-situ* gel, and 3) the BMP-2 suspended in the prepared *in-situ* gel base. Rabbits were sacrificed at 1, 3, 6, 12, and 24 h and at 3, 7, and 14 days following the injection. The maxilla was dissected and the skin tissue removed and wrapped. The gel remaining under the maxillary skin was assessed after the obtained clean gel was weighed and dissolved using anhydrous methanol with a volume equal to five times the gel’s weight by ultrasound. The BMP-2 content was allocated as mentioned in previous sections of this paper. Finally, the percentage of BMP-2 released *in vivo* was estimated and a corresponding content‒time curve percentage was constructed to determine the *in-vivo* release rate of the BMP-2 from the fabricated nano *in-situ* gel.

## 3 Results and discussion

### 3.1 Evaluation of the BMP-2‒loaded NE

The developed NEs were found to be homogenous and almost clear, with no signs of precipitation.

#### 3.1.1 Determination of BMP-2‒loaded NE’s droplet size

One of the most important parameters to be examined for the successful characterisation of the produced formulations is the droplet size of nanoemulsions (Quiroz-Segoviano et al., 2019). The NEs had a droplet size of between 88 ± 1.0 and 167 ± 3.5 nm, as summarized in Table 2, and a polydispersity index of 0.11–0.35. These results showed the good stability, uniformity, and size of the BMP-2‒loaded NE. A cubic model of polynomial analysis disclosed the best considerable mean squared value surpassing the residual error (*p* < .0001), so it was assumed for the analysis of the data of the NE size. The proposed statistical design revealed the model’s adequacy for considering the impact of the BMP-2 aqueous solution level (A), Lauroglycol FCC level (B), and Labrafac PG oil level (C) on the BMP-2-NEs’ droplet size. The model had an adjusted *R* ^2^ value of 0.9995, which was in close proximity to the expected *R* ^2^ value of 0.9904. The analysis of variance (ANOVA) of the gathered data gave rise to the equation below.

$$\begin{array}{ll} Droplet\;Size & =‒\;3832.05\;A+293.93\;B+171.41\;C+6535.13\;AB\\ & +6971.88\;AC\;‒\;545.82\;BC\;‒\;5451.22\;ABC\\ & +4111.79\;AB\left(A‒B\right)\\ & +3397.71\;AC\left(A‒C\right)\;‒\;415.47\;BC\left(B‒C\right) \end{array}$$ (2)

**TABLE 2:** I-optimal exchange coordinate design and responses of BMP-2‒loaded NEs.

Run	A: BMP-2 aqueous solution level	B: Lauroglycol™ FCC surfactant level	C: Labrafac PG oil level	Y_1_: Globule size (nm)	Y_2_: Stability index (%)	PDI
1	0.166	0.200	0.633	161 ± 1.4	63 ± 1.5	0.21
2	0.100	0.301	0.598	121 ± 2.1	84 ± 2.2	0.25
3	0.144	0.254	0.601	149 ± 4.1	75 ± 1.7	0.19
4	0.184	0.400	0.415	88 ± 1.0	96 ± 3.5	0.11
5	0.200	0.225	0.574	143 ± 2.8	71 ± 2.6	0.24
6	0.153	0.376	0.469	92 ± 2.0	92 ± 4.1	0.35
7	0.100	0.223	0.676	167 ± 3.5	70 ± 1.1	0.33
8	0.141	0.310	0.548	115 ± 4.5	87 ± 2.5	0.14
9	0.166	0.200	0.633	160 ± 1.9	64 ± 3.3	0.27
10	0.141	0.310	0.548	114 ± 0.5	87 ± 2.9	0.32
11	0.200	0.276	0.523	130 ± 2.7	81 ± 1.3	0.16
12	0.200	0.325	0.474	99 ± 1.8	90 ± 3.9	0.28
13	0.141	0.310	0.548	115 ± 2.2	88 ± 2.4	0.30
14	0.100	0.400	0.500	101 ± 3.1	94 ± 3.9	0.12

**FIGURE 1:**
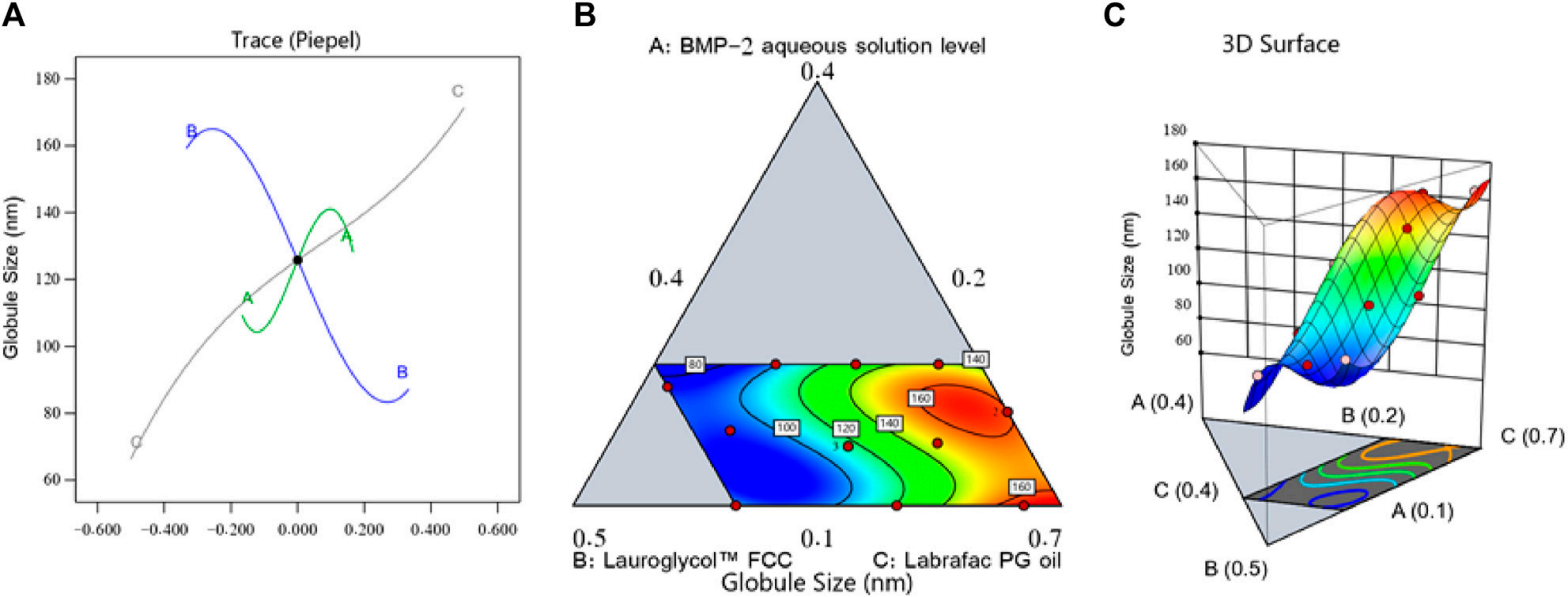
Perturbation outline **(A)**, contour diagram **(B)**, and 3D surface graph **(C)** showing the effects of different factors on the droplet size (Y_1_) of different BMP-2-NE formulations.

Figure 1 shows the perturbation, contour, and three-dimensional (3D) surface plots that revealed the effect of the components on the droplet size of the BMP-2-NEs. As can be seen in the figure, factor A (i.e., the BMP-2 aqueous solution level) exerted a positive effect on the droplet size, and, therefore, it would be expected that increasing the concentration of the BMP-2 aqueous solution would also increase the size of the droplets. Such results might be due to the higher expected size of the internal phase droplets when the concentration of protein aqueous solution was increased (Hassan et al., 2015). Moreover, the increase in the concentration of the surfactant (i.e., factor B) yielded a corresponding decrease in droplet size. This could be due to the amphiphilic nature of factor B. Lauroglycol FCC is a non-ionic surfactant and hence has the potential to lower the interfacial tension between the organic and aqueous phases, and this would lead to the downsizing of the droplets of the NE’s internal phase (Hosny et al., 2020). Additionally, the increase in the surfactant level would increase the firmness of the surfactant film that developed around the NE droplets and hence improve their stability and prevent their aggregation (Hosny et al., 2021b). In the meantime, the increase in the Labrafac PG oil level (i.e., factor C) resulted in a considerable increase in the droplet size. This finding could be due to the decrease in the surfactant level that accompanied the increase in the oil level, which would diminish the surfactant’s capacity to reduce the size of the droplets and decrease their stability, leading to more aggregation and larger droplets (Hosny et al., 2020).

#### 3.1.2 Determination of BMP-2‒loaded NE stability index

The BMP-2‒loaded NEs had a stability index that ranged from 70% ± 1.1% to 96% ± 3.5%, as shown in Table 2. A quadratic model of polynomial analysis offered the greatest considerable average squared value that outweighed the residual error (*p* < .0001), so it was suggested for analyzing the stability index data. The statistical design allowed the chosen model to explain the notable impact of the BMP-2 aqueous solution level (A), Lauroglycol FCC level (B), and Labrafac PG oil level (C) on the BMP-2-NEs’ stability index. The proposed model had an adjusted *R* ^2^ value of 0.9892, which was near the expected *R* ^2^ value of 0.9809. An ANOVA of the stability index data provided the equation below.

Stability Index=+71.81 A+90.81 B+63.69 C+52.76 AB ‒ 11.81 AC +53.73 BC  (3)

**FIGURE 2:**
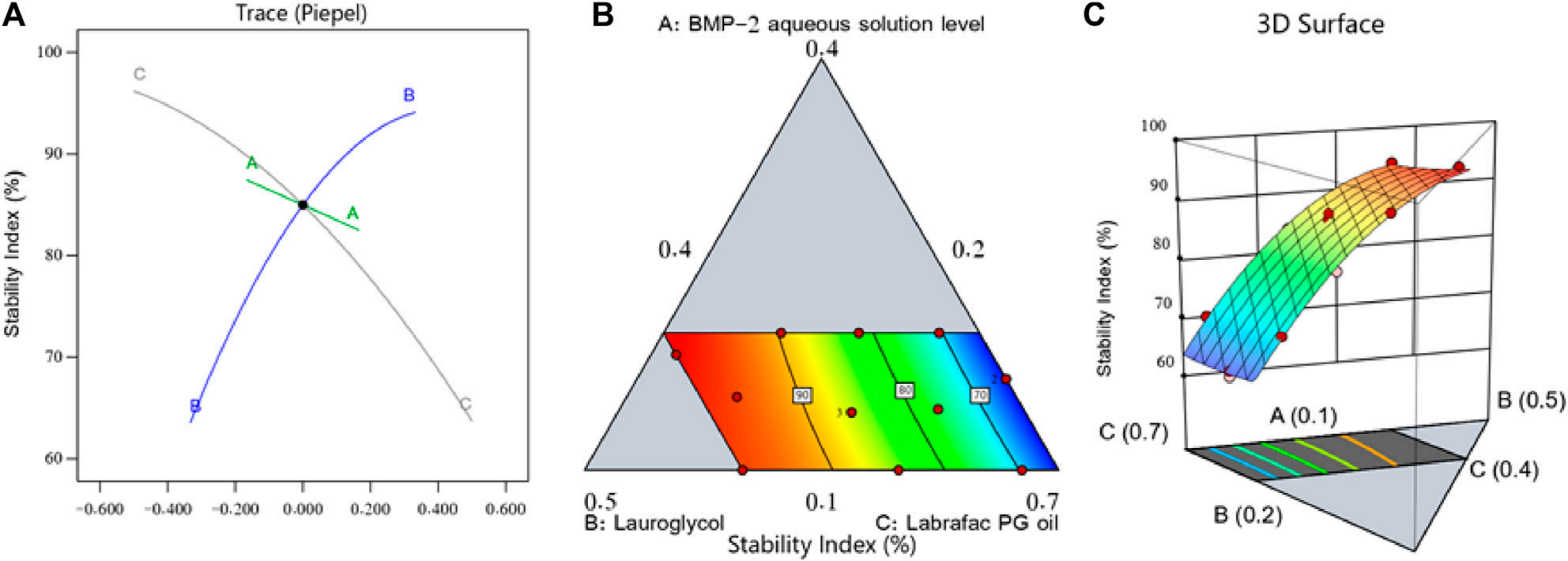
Perturbation diagram **(A)**, contour graph **(B)**, and 3D surface graph **(C)** showing the influence of various factors on the stability index (Y_2_) of different BMP-2-NEs.

Figure 2 shows the main effect graph, contour diagram, and 3D surface diagram, which reveal the impact of the factors on the BMP-2-NEs’ stability index. As per Figure 2, the BMP-2 aqueous solution level (i.e., factor A) exerted a negative impact on the stability index, and therefore, increasing the BMP-2 aqueous solution concentration would decrease the NE’s stability. Such an effect could be due to the expected increase of the NEs’ droplet size when the protein aqueous solution concentration was increased. Hence, according to Stock’s law, there is a faster sedimentation of the internal phase globules, leading to decreased stability (Hosny et al., 2021b). On the other hand, the increase in the surfactant concentration (i.e., factor B) led to a corresponding increase in the stability index. This could be explained by the accompanying decrease in particle size, which would decrease the globules’ sedimentation rate. Additionally, the increase in the surfactant level would increase the firmness of the surfactant film that had developed around the internal phase droplets and, hence, improve the droplets’ stability and prevent their aggregation (Hosny et al., 2021a). In parallel, the increase in the Labrafac PG oil level (i.e., factor C) led to a significant decrease in the stability index. Such a decrease in the stability of the NEs could be due to the decrease in the surfactant level that accompanied the increase in the oil level; this would decrease the capacity of the surfactant to reduce the droplets’ size and decrease their stability, leading to more aggregation and resulting in larger droplets with a faster sedimentation rate and, hence, decreased stability (Hosny et al., 2021c).

### 3.2 Optimization and evaluation of NE formulations

**FIGURE 3:**
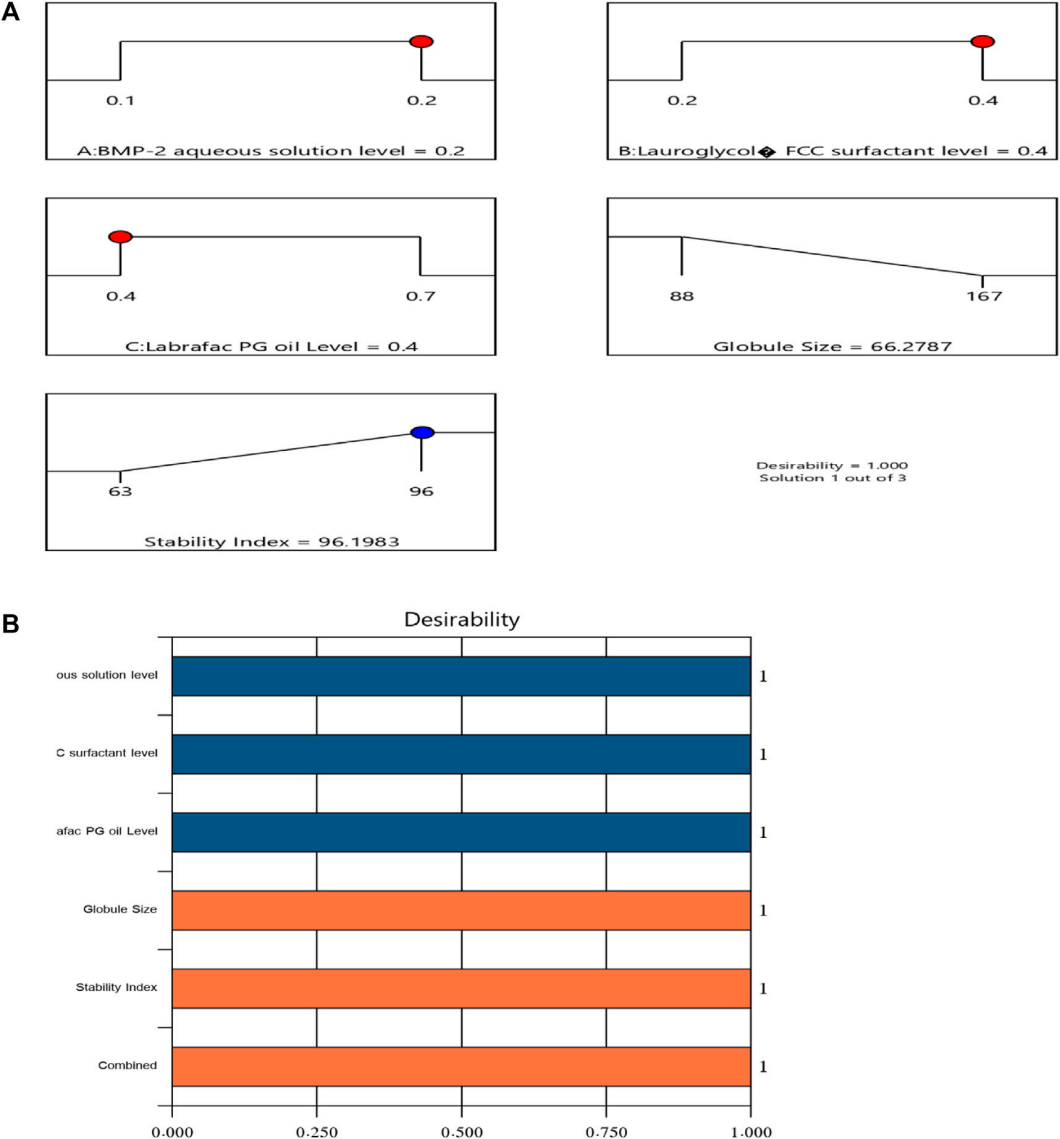
Bar chart and desirability ramp for optimization. **(A)** The levels of the independent variables are displayed along with the anticipated values for the responses in the desirability ramp. **(B)** The bar graph displays the values for desirability for the total responses.

After completing the above investigations, an optimal NE formulation with the most reasonable characteristics was revealed. Many combinations of factors had been generated with the chosen design. The optimal formulation contained 0.2% of the BMP-2 aqueous solution, 0.4% of the Lauroglycol FCC, and 0.4% of the Labrafac PG oil and had a desirability value of 1.000. The optimal BMP-2-NE had a droplet size of 68 ± 2.0 nm and a stability index of 96% ± 1.3%. These findings were very close to the expected values for these factors, which were 66.279 nm for the droplet size and 96.198% for the stability index. Figure 3 sets forth the desirability ramp and bar chart for changing levels of the independent variables and the expected measured responses of the optimal formulation.

### 3.3 Characterization of the optimized BMP-2-NE‒loaded *in-situ* gel

#### 3.3.1 Viscosity measurement

**FIGURE 4:**
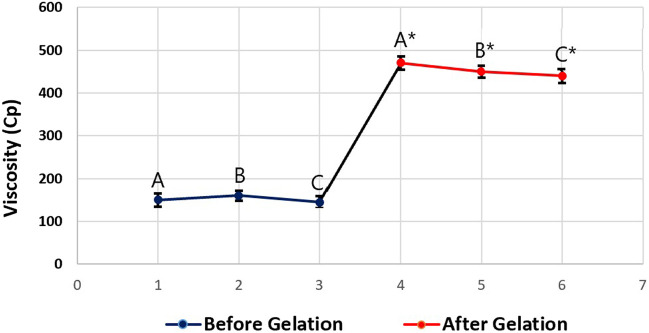
Viscosity values of the tested optimal formulation before gelation (A, B, and C) and after gelation (A*, B*, and C*).

The viscosity of the tested PPSG sample was measured three times before and after gelation. The average viscosity was 150 ± 15 Cp before gelation and 450 ± 10 Cp after gelation. These results showed the nature of the *in-situ* gelation of the formulation. Figure 4 illustrates the change in viscosity caused by the solvent exchange mechanism when PBS replaced ethanol in the formulation and induced gelation. This action mimicked the action that was expected to happen *in-vivo* upon injection of the formulation into the maxilla. Comparable results were found in the literature (Srichan and Phaechamud, 2017).

#### 3.3.2 Measurement of gel strength

When fabricating an insertable PPSG, the gel strength is a paramount characteristic to study. The quixotic strength of a fabricated gel encourages the easy insertion of a solution of an active ingredient and should result in no seepage at the insertion site. It is important for any gel base to have a reasonable gel strength. Formulations having a gel strength of less than 25 s might be subject to leakage from an injection site because they might lack the required structural integrity. Alternatively, gel bases with a strength higher than 50 s might be too robust and uncomfortable for patients. The gel vigor of the prepared samples after the addition of 30% PBS was 41 s, and this could be considered suitable for an injectable *in-situ* gel. Previously published work reported similar results (Hassan et al., 2016).

#### 3.3.3 Water absorption

Using miscible solvents such as water and ethanol provides a good base for the fast sedimentation of phospholipids and *in-situ* gel phase separation. Ethanol is thought to diffuse out of the *in-situ* gel formulation upon contact with an aqueous solution, while the aqueous solution will permeate the gel base. The solvent diffusion rate is a paramount parameter impacting the BMP-2 phase transition and release from the gel (Hettiaratchi et al., 2018). It was observed that the *in-vitro* water absorption through the *in-situ* gel base was fastest during the first 2 h. More than 10% of the water absorption occurred within 2 min following dialysis, and the highest water absorption percentage of the tested gel base was 45% ± 4%.

#### 3.3.4 *In-vitro* release behavior of optimal formulation

**FIGURE 5:**
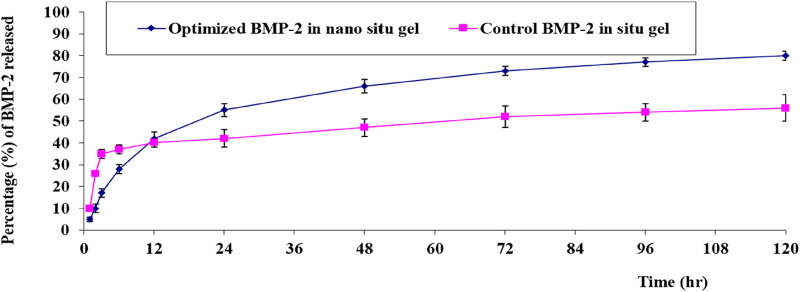
*In-vitro* release behavior of BMP-2 aqueous solution from the optimal formulation of the loaded gel compared with the control BMP-2 solution of the loaded gel (mean ± SD, *n* = 3).

The *in-vitro* release of the BMP-2 from the optimal NE *in-situ* gel formulation and the control gel fabricated by solubilizing the BMP-2 solution in the gel base had the following results: gelation occurred within 3 h, 37% ± 3% was released within 12 h, and only 53% ± 6% was released at the end of the experiment. For the optimal gel, in which gelation occurred within 2 h, 42% ± 1% of the BMP-2 was released within 12 h and 82% ± 2% was released at the end of the test. Figure 5 illustrates the consistent and prolonged release of BMP-2 from the optimal formulation of the loaded gel with a minimum initial burst release compared with the control gel. Also, the optimal formulation had a higher percentage of release, a lower standard deviation (SD), and, overall, a more enhanced release profile.

#### 3.3.5 *In-vivo* studies

After reviewing the data collected from the *in-vivo* experiment, the following findings were obtained.

In an animal group that received the hydrogel developed by solubilizing BMP-2 in 1% hydroxypropyl cellulose solution, the gel was absorbed and diffused away from the injection site within approximately 6 h (i.e., approximately 30% of the BMP-2 was released from the gel during the first hour and 80% within 3 h; after 6 h, no gel was detected at the site of injection). For the control treated with PPSG-loaded BMP-2 aqueous solution, only approximately 30% of the BMP-2 was released during the first hour and 65% within 3 h; after 6 h, 22% of the BMP-2 was still entrapped in the gel base. This indicated that the transitioning of the base into a gel occurred at 3–6 h, that the release occurred in a controlled manner, and that the gel was completely dissolved and absorbed by tissues after 168 h (7 days). For the group treated with the optimal BMP-2-NE‒loaded PPSG, almost 15% of the BMP-2 was released after the first hour and 39% within 3 h; this indicated that the base transitioned into a gel within 1–3 h and that after 6 h, nearly 56% of the BMP-2 was still entrapped within the gel base (i.e., 44% of the BMP-2 was released). This indicated that the release had occurred in a controlled manner and continued in this manner for 336 h (14 days). After this time, 8% of the BMP-2 was still entrapped and the base had not completely dissolved.

**FIGURE 6:**
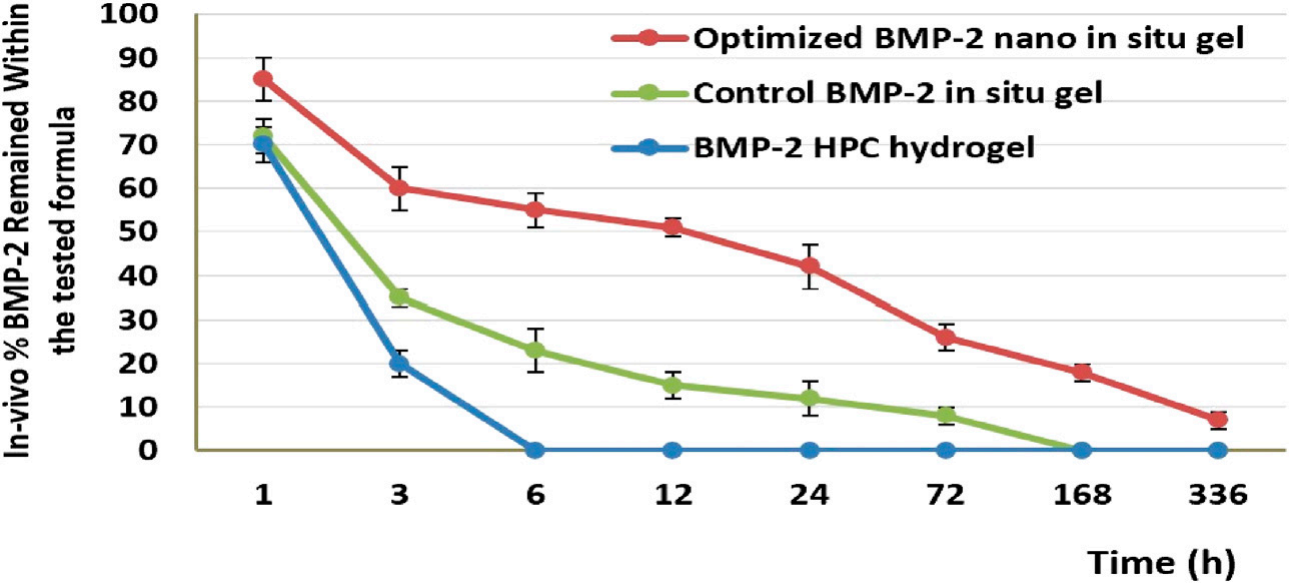
The *in-vivo* release percentage of BMP-2 in the maxilla for different treatment groups after 14 days.

The preceding results illustrated the superior activity of the PPSG loaded with the optimal formulation in treating the major congenital malformation CLP. The formulation could be administered easily as a liquid and then become a gel, in which form it was localized at a site for the longest period. In addition, the *in-situ* gel formulation had a prolonged and controlled release of the active agent when in the gel state owing to the cross-linked structure of the gel base. This offered the maximal benefits from the administered dosage since it would persist at the site for a long time and, hence, might prevent the need for surgical intervention to correct the CLP. Similar outcomes were previously reported (Camargo et al., 2013; Hassan et al., 2016). Figure 6 shows the percentage of BMP-2 that remained within the maxilla for 14 days for different treated groups.

## 4 Conclusion

Incorporating an NE of BMP-2 within a PPSG is a delivery paradigm that appears to be a new and superior noninvasive method of restoring the alveolar cleft defects that are one of the most common congenital deformations. The animal group treated with the PPSG loaded with the optimal BMP-2-NE enjoyed a controlled and prolonged release of BMP-2 within the maxilla for 14 days. This indicates that the system is a beneficial way of delivering this important protein and is an easy, less painful, and safe procedure. This novel procedure has many future implementations. It could be a substitute for surgical interventions of the maxilla or an early grafting method for treating CLP patients.

## Data Availability

The raw data supporting the conclusion of this article will be made available by the authors, without undue reservation.
